# The Voxel-Wise Functional Connectome Can Be Efficiently Derived from Co-activations in a Sparse Spatio-Temporal Point-Process

**DOI:** 10.3389/fnins.2016.00381

**Published:** 2016-08-23

**Authors:** Enzo Tagliazucchi, Michael Siniatchkin, Helmut Laufs, Dante R. Chialvo

**Affiliations:** ^1^Institute for Medical Psychology, Christian-Albrechts UniversityKiel, Germany; ^2^Department of Neurology and Brain Imaging Center, Goethe University Frankfurt am MainGermany; ^3^Department of Sleep and Cognition, Netherlands Institute for NeuroscienceAmsterdam, Netherlands; ^4^Department of Neurology, University Hospital Schleswig-Holstein, Christian-Albrechts-University KielKiel, Germany; ^5^Consejo Nacional de Investigaciones Científicas y Tecnológicas (CONICET)Buenos Aires, Argentina; ^6^Center for Multidisciplinary Complex Systems Studies and Brain Sciences (CEMSC3), Escuela de Ciencia y Tecnología, Universidad Nacional de San MartínBuenos Aires, Argentina

**Keywords:** functional connectome, functional connectivity, dimensionality reduction, point process, resting state fMRI

## Abstract

Large efforts are currently under way to systematically map functional connectivity between all pairs of millimeter-scale brain regions based on large neuroimaging databases. The exploratory unraveling of this “functional connectome” based on functional Magnetic Resonance Imaging (fMRI) can benefit from a better understanding of the contributors to resting state functional connectivity. In this work, we introduce a sparse representation of fMRI data in the form of a discrete point-process encoding high-amplitude events in the blood oxygenation level-dependent (BOLD) signal and we show it contains sufficient information for the estimation of functional connectivity between all pairs of voxels. We validate this method by replicating results obtained with standard whole-brain voxel-wise linear correlation matrices in two datasets. In the first one (*n* = 71), we study the changes in node strength (a measure of network centrality) during deep sleep. The second is a large database (*n* = 1147) of subjects in which we look at the age-related reorganization of the voxel-wise network of functional connections. In both cases it is shown that the proposed method compares well with standard techniques, despite requiring only data on the order of 1% of the original BOLD signal time series. Furthermore, we establish that the point-process approach does not reduce (and in one case increases) classification accuracy compared to standard linear correlations. Our results show how large fMRI datasets can be drastically simplified to include only the timings of large-amplitude events, while still allowing the recovery of all pair-wise interactions between voxels. The practical importance of this dimensionality reduction is manifest in the increasing number of collaborative efforts aiming to study large cohorts of healthy subjects as well as patients suffering from brain disease. Our method also suggests that the electrophysiological signals underlying the dynamics of fMRI time series consist of all-or-none temporally localized events, analogous to the avalanches of neural activity observed in recordings of local field potentials (LFP), an observation of potentially high neurobiological relevance.

## Introduction

The human brain comprises an interconnected network of cortical and sub-cortical regions globally linked by anatomical long-range tracts of connections. The mapping of the corresponding functional connections at a particular spatial scale (termed connectome in contemporary neuroscience; Sporns et al., 2004; Sporns, 2011) is an important ingredient in the process of understanding how the human brain can perform diverse cognitive functions. Furthermore, many neurological and psychiatric diseases can be understood in terms of deviations from a healthy connectome (Fox and Greicius, 2010; Kelly et al., 2012).

Advances in neuroimaging methods, such as Diffusion Tensor Imaging (DTI) and Diffusion Spectrum Imaging (DSI) allow the *in vivo* mapping of the human structural connectome at a large-scale (Hagmann et al., 2008). Blood oxygenation level-dependent (BOLD) functional Magnetic Resonance Imaging (fMRI) allows for a functional counterpart of the anatomical connectome, a notion first introduced about a decade ago (Sporns et al., 2004; Eguiluz et al., 2005; Salvador et al., 2005) by computing the statistical covariance between all pairs of BOLD signals. This functional connectome contains information on how all pairs of regions (at a certain spatial scale) relate dynamically and collectively with each other.

These two approaches are being applied by international coordinated efforts to systematically map connectomes in very large populations of subjects and at the highest temporal and spatial resolution currently available (see for instance Biswal et al., 2010; Smith et al., 2013; Van Essen et al., 2013). These efforts will eventually lead to the availability of large-scale databases useful to account for potential inter-subject variability caused by different demographical variables, as well as to reduce the harmful effect of noise and artifacts through massive averaging.

These collaborative efforts need to be paralleled by methodological developments facilitating efficient extraction of relevant information from the data. Common strategies are based on averaging BOLD signals over brain parcellations comprising extended regions, thus reducing the dimensionality of the problem as well as the number of required computations. However, there are several problems inherent to this approach. First, all detail of the functional connectome inside each region of the parcellation is lost. Second, partitions are usually arbitrary and therefore might sub-divide a functionally coherent region into many regions. Different studies have addressed how the properties of parcellation-based networks can change depending on region selection (Wang et al., 2009; Zalesky et al., 2010). Third, efforts to increase the spatial resolution of fMRI sequences are pointless if data will be down-sampled after acquisition by averaging BOLD signals inside a small number of regions in a parcellation.

The objective of this paper is to show how a very sparse representation of brain activity, namely a discrete spatio-temporal point-process, is able to estimate the whole brain voxel-wise functional connectome. This point-process is derived from the times at which the BOLD signals reach some maximum level of activity, either by detecting crossings of an arbitrary threshold, or by the identification of local peaks, i.e., the point-process comprises large amplitude events in the data. At its core, our proposed method is based on identifying a basis of discrete contributions to resting state fMRI signals, in analogy to other neural recording modalities (such as spikes in intra- and extra- cellular recordings). Following this analogy, once the relevant events are identified, much of the signal (i.e., the stereotypical response associated with a discrete event) can be disregarded without reducing their information content, facilitating data storage, manipulation and interpretation (this analogy is limited, however, since neural recordings provide more sampling points than fMRI recordings and hence a larger number of discrete events). The main merit of this method is to reduce the continuous representation of BOLD signals into a series of timings associated with events of interest, thus (1) drastically reducing the dimensionality of the data, (2) abstracting the relevant information from sources of noise.

It has been shown previously that this method suffices to reproduce large-scale patterns of coordinated activity (Tagliazucchi et al., 2011, 2012a) termed Resting State Networks (RSN; Beckmann et al., 2005) and is essentially identical to the de-convolution of the signals as a series of discrete impulse functions (Petridou et al., 2013). Furthermore, de-convolution into a point-process can lessen the impact of hemodynamic lags for the estimation of causality between BOLD signals (Wu et al., 2013). Here we contribute a systematic evaluation of the capacity of this method to reproduce *all* bivariate relationships between signals (i.e., whole-brain correlation matrices). This validation is obtained, for the first time, from a large database of subjects *n* = 1147) scanned with different parameters at different locations, thus supporting its universal validity.

We also investigated whether abstracting the signal into a point-process could yield benefits from the perspective of reducing confounds and noise in the data. For this we adopted a practical, classification-based approach, investigating how accurately connectivity matrices derived from the point-process and from linear correlations could distinguish two groups of subjects (younger and older subjects from the *n* = 1147 database). We hypothesized that keeping high-amplitude events in the data could disregard low-amplitude noise and result in a better classification accuracy than the one obtained using full BOLD signals.

## Materials and methods

**Figure 1:**
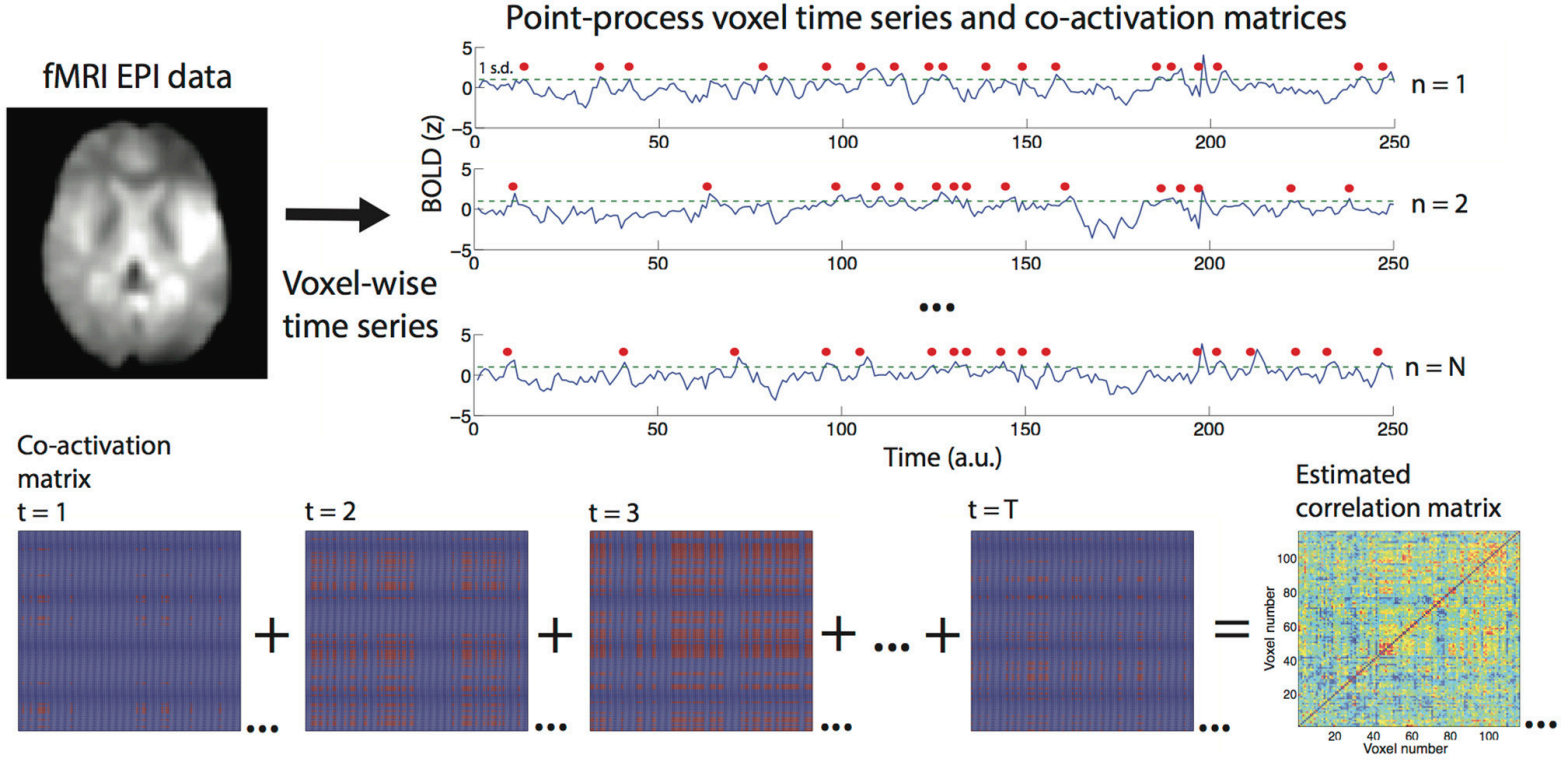
**Procedure to construct the point-process and to estimate functional connectomes**. For every voxel, signals are converted to *z*-scores and a discrete event marked after every threshold crossing (in this example the threshold was set to 1 standard deviation, crossings are marked with a red dot). For every volume a whole brain co-activation matrix is derived, and the sum of all co-activation matrices estimates the functional connectivity matrix or correlation matrix (only a fraction of the matrices are shown in this example).

We will first describe all steps of the proposed method and then introduce different datasets used for validation as well as to show possible applications. The general procedure followed to estimate correlation networks via the point-process analysis is graphically outlined in Figure 1.

### Voxel-wise correlation matrix

Consider an fMRI measure consisting of N voxels and T volumes, represented as F_n_(t), with 0 ≤ n ≤ N and 0 ≤ t ≤ T. Thus, F_n_(t) represents the BOLD signal at voxel n and time t. The common definition of voxel-wise correlation matrix (Eguiluz et al., 2005) is as follows,

(1) $$\begin{array}{c} {\text{R}}_{\text{ij}}=\frac{<({\text{F}}_{\text{i}}\;-<{\text{F}}_{\text{i}}>)({\text{F}}_{\text{j}}-<{\text{F}}_{\text{j}}>)>}{\sigma ({\text{F}}_{\text{i}})\sigma ({\text{F}}_{\text{j}})} \end{array}$$

where < F_i_> and σ(F_i_) represent the mean value and the standard deviation of the BOLD signal at the voxel i, respectively. Note that according to this definition, for the computation of R_ij_, Equation (1) must be evaluated N(N−1)∕2 times (although not serially in efficient implementations). Often these calculations are used to define functional connectivity networks which in turn allow for further analysis of the resulting graphs.

### Constructing the point-process

The approach here proposed starts with converting the BOLD signal at every voxel into its z-score, ${\tilde{\text{F}}}_{\text{i}}=\frac{{\text{F}}_{\text{i}}\;-\;<{\text{F}}_{\text{i}}>}{\sigma \left({\text{F}}_{\text{i}}\right)}$. This is done under the assumption that, according to our formalism, the absolute amplitude of the BOLD signal carries less information than its temporal evolution (for the biological underpinnings of this assumption please see the Discussion section). To define the point-process, the *a priori* arbitrary threshold γ is selected and the spatio-temporal process PP_i_(t)) is defined as follows:

(2) $$\begin{array}{c} {\text{PP}}_{\text{i}}(\text{t})=\left\{ \begin{array}{l} 1\;\;\text{if}\;{\tilde{\text{F}}}_{\text{i}}\left(\text{t}\right)\;<\;\;\gamma \;\text{ and }\;{\tilde{\text{F}}}_{\text{i}}\left(\text{t}+1\right)\;>\;\;\gamma \\ \;0\;\text{otherwise} \end{array}\right. \end{array}$$

This point-process was introduced in a previous publication (Tagliazucchi et al., 2012a) where we showed that it suffices to replicate the topographical features of the major canonical RSN, even though for most values of t and i, PP_i_(t)) will be zero (indeed, taking γ = 1, for a signal of T = 240 on average the point-process is non-zero for 15±3 time points, or approximately 6% of the data—see Tagliazucchi et al., 2012a). Note that once the point-process is constructed much of the data can be discarded. From a signal comprising 240 values, only a series of (on average) 15 numbers needs to be retained, namely, the timings of the events in the point-process. Clearly, this results in a considerable compression of the fMRI data.

Alternatively, PP_i_(t) can be defined by the (high amplitude) local peaks of the BOLD signal. For this, BOLD signals are also converted to z-scores and all sufficiently large peaks (for instance, those above an arbitrary threshold) are the points represented in PP_i_(t). The formal definition is as follows,

(3) $$\begin{array}{c} {\text{PP}}_{\text{i}}(\text{t})\;=\text{​ }\left\{ \begin{array}{l} 1\;\text{if}\;{\tilde{\text{F}}}_{\text{i}}(\text{t})\;>{\tilde{\text{F}}}_{\text{i}}\left(\text{t }-\;1\right)\;\text{ and}\;\;{\tilde{\text{F}}}_{\text{i}}\left(\text{t}\right)>\;{\tilde{\text{F}}}_{\text{i}}\;\left(\text{t }+\;1\right)\text{ and}\\ \;{\tilde{\text{F}}}_{\text{i}}\left(\text{t}\right)>\;\gamma \\ \;0\;\text{ otherwise}\; \end{array}\right. \end{array}$$

Although formally both methods are justified, it will be shown later that either definition of the point-process leads to similar results.

### Estimating correlations from the point-process

After converting F_i_ into PP_i_(t) we introduce the following framework to generalize the methods introduced in Tagliazucchi et al. (2012a), from the estimation of seed based correlations to the efficient computation of all pairs of correlations between voxels. We first define the co-activation matrices A_ij_(t) as follows:

(4) $$\begin{array}{c} {\text{A}}_{\text{ij}}\left(\text{t}\right)=\;{\text{PP}}_{\text{i}}\left(\text{t}\right){\text{PP}}_{\text{j}}(\text{t}) \end{array}$$

Note that according to this definition, A_ij_(t) only has two possible values: A_ij_(t) = 1 if at time t the point-process is non-zero both at voxels i and j, and A_ij_(t) = 0 otherwise.

The co-activation matrices defined in Equation (4) can be used to estimate the functional connectivity between all pairs of voxels in the brain by performing a simple matrix addition. Two highly synchronized signals will cross the threshold together most of the time, thus a measure of coupling between the signals can be obtained by counting the number of times the signals crossed the threshold together. This is formalized simply by,

(5) $${\text{C}}_{ij}\;=\;\sum_{\text{t=1}}^{\text{T}}{\text{A}}_{\text{ij}}\;(\text{t})\;=\;\sum_{\text{t=1}}^{\text{T}}{\text{PP}}_{\text{i}}\;(\text{t})\;{\text{PP}}_{\text{j}}(\text{t})$$

In matrix notation, this can be succinctly summarized as C = PP PP^T^, considering PP as a matrix with voxels as rows and time as columns and containing the point-process. The matrix C_ij_ contains in its i, j entry the number of shared co-activations between BOLD signals at voxels i and j. Note that since all A_ij_ are symmetrical matrices, then C_ij_ is also symmetrical. Note also that the matrices A_ij_(t) contain valuable information about instantaneous co-activations between voxels and as such their analysis might be important to understand the temporal evolution of large-scale synchronization between brain regions (Tagliazucchi et al., 2012b; Hutchison et al., 2013).

The main issue with this matrix as a measure of functional connectivity is that it is not normalized, therefore there is no way to directly decide (for instance) if a perfect synchronization between signals has been reached. An appropriate normalization for this matrix would be as follows,

(6) $$\begin{array}{c} {\tilde{\text{C}}}_{\text{ij}}=\frac{{\text{C}}_{\text{ij}}}{\text{max}\left(\sum_{\text{t }=\;0}^{\text{T}}\text{P}{\text{P}}_{\text{i}},\;\sum_{\text{t }=\;0}^{\text{T}}\text{P}{\text{P}}_{\text{j}}\right)}=\frac{{\text{C}}_{\text{ij}}}{\text{max}\left({\text{C}}_{\text{ii}},{\text{C}}_{\text{jj}}\right)} \end{array}$$

This definition of ${\tilde{\text{C}}}_{\text{ij}}$ is reasonable since C_ij_ achieves its highest possible value if all threshold crossings are also shared between both voxels. However, one voxel could have all its threshold crossings in common with the other, whereas the opposite might not be true (since the other voxel could have a larger number of crossings in total (this can be the case only if C_ii_ ≠ C_jj_), thus normalizing using the maximum between the number of crossings at both voxels is required. Also, ${\tilde{\text{C}}}_{\text{ij}}$ is symmetrical with this normalization.

The normalization presented in Equation (6) requires the maximum value between the numbers of threshold crossings at all pairs of voxels. If normalization is needed, then a more efficient approximate solution is to divide by the number of threshold crossings without taking the maximum value, for instance, across rows or columns of the matrix, and then symmetrizing (if needed) the result by averaging with the transpose:

(7) $$\begin{array}{c} {\tilde{\text{C}}}_{\text{ij}}=\frac{1}{2}\left[\frac{{\text{C}}_{\text{ij}}}{{\text{C}}_{\text{ii}}}\;+\;\frac{{\text{C}}_{\text{ji}}}{{\text{C}}_{\text{ii}}}\right] \end{array}$$

Note that $\frac{{\text{C}}_{\text{ij}}}{{\text{C}}_{\text{ii}}}$ deviates from a symmetrical matrix only in the case of different numbers of threshold crossing between voxels (C_ii_ ≠ C_jj_). Note also that normalization might not be necessary if comparing fixed-length recordings between two populations, under the assumption that the rate of events in the point-process is not different between groups.

For the computation of ${\tilde{\text{C}}}_{\text{ij}}$ all steps can be performed efficiently in vectorized form in any language with matrix manipulation capabilities (for instance, MATLAB or Python with NumPy), in particular, after constructing the point-process in Equation (2), the operations involved consist of a single matrix multiplication (Equations 4 and 5), multiplication by scalars and matrix symmetrization (Equation 7). In this work, all computations were performed using a 8 core CPU running at 2400 MHz with a total of 128 GB built-in memory.

After introducing the core methods, we now discuss the methodology for the validation of our results.

### Measures derived whole brain voxel-wise correlations used for method validation

The number of connections derived in a voxel-wise analysis complicates easy visualization of networks and their changes across conditions. Thus, in the many applications of functional connectomes found in the literature, rarely whole-brain voxel-wise networks are directly visualized. Instead, lower-dimensional metrics are to be derived, which are easy to visualize as 3D maps overlaid on brain anatomy. One possible choice is to assess measures of network centrality, this is, how important nodes are in the network, thus collapsing all connections attached to a node into a single number. A straightforward definition in a weighted network is the strength (Barthelemy et al., 2005), defined as:

(8) $$\begin{array}{c} {\text{S}}_{\text{i}}=\sum_{\text{j}=1}^{\text{N}}{\text{R}}_{\text{ij}} \end{array}$$

In the present case, using the point-process to estimate correlations, R_ij_ is replaced by ${\tilde{\text{C}}}_{\text{ij}}$. Nodes with the highest strength values are termed hubs and their reorganization has been repeatedly linked to different brain pathologies (Crossley et al., 2014), such as coma (Achard et al., 2012) or Alzheimer's disease (Buckner et al., 2009).

Note that the evaluation of Equation (8) requires the whole brain correlation network. In the case of a voxel-wise network, centrality of nodes (i.e., voxels) can be easily visualized as a 3D map overlaid on an anatomical image.

Another measure employed for validation of our method is the interhemispheric or homotopic connectivity. This is defined as the correlation between the BOLD signal of every voxel and the contralateral voxel. Interhemispheric connectivity is in particular useful to quantify re-organization of functional connectomes for which left-right asymmetries are expected (as in the case of aging, see Dolcos et al., 2002).

### Datasets

To demonstrate the validity of our proposal two different datasets from previously published studies will be used. The first dataset comprises BOLD fMRI recordings from the 1000 Functional Connectomes database, and the second dataset comprises recordings from a recently published study in which combined EEG, EMG, BOLD-fMRI, and physiological data were obtained from 71 subjects.

The Connectome dataset was downloaded from the 1000 Functional Connectome Project online database (*http://fcon_1000.projects.nitrc.org*). Demographics, scanning parameters, and experimental conditions are described in the database website as well as in Tagliazucchi and Laufs (2014). Only epochs of wakefulness were employed in the present analysis. For more information on sleep vs. wakefulness classification in this dataset (see Tagliazucchi et al., 2012c; Tagliazucchi and Laufs, 2014). Since individual data presents variable length in this data set, normalization (Equation 7) was always required.

Data from a previously published study (Tagliazucchi and Laufs, 2014) was used for the sleep dataset. A total of 71 subjects were selected from a larger dataset on the basis of successful multimodal polysomnographic data recording and quality (written informed consent, approval by the local ethics committee). All subjects were scanned during the evening and instructed to close their eyes and lie still and relaxed. A group of 55 subjects was formed out of the original dataset of 71 subjects by excluding subjects who did not fall asleep. Hypnograms obtained via expert sleep staging based on AASM rules (American Academy of Sleep Medicine, 2007) were scanned for contiguous epochs of wakefulness, N1, N2, and N3 sleep lasting 250 volumes (~ 2 min), resulting in 84 epochs of wakefulness, 16 epochs of N1 sleep, 19 epochs of N2 sleep, and 20 epochs of N3 sleep. Sleep epochs are present (by construction) fixed length in this data set (250 volumes), therefore normalization (Equation 7) was not required under the assumption that sleep does not modify the rate of points in the data.

EEG was recorded via a cap (modified BrainCapMR, Easycap, Herrsching, Germany) during fMRI acquisition (1505 volumes of T2^*^-weighted echo planar images, TR/TE = 2080/30 ms, matrix 64 × 64, voxel size 3 × 3 × 2 mm^3^, distance factor 50%; FOV 192 mm^2^) at 3 T (Siemens Trio, Erlangen, Germany) with an optimized polysomnographic setting [chin and tibial EMG, ECG, EOG recorded bipolarly (sampling rate 5 kHz, low pass filter 1 kHz), 30 EEG channels recorded with FCz as the reference (sampling rate 5 kHz, low pass filter 250 Hz), and pulse oxymetry, respiration recorded via sensors from the Trio (sampling rate 50 Hz)] and MR scanner compatible devices (BrainAmp MR+, BrainAmp ExG; Brain Products, Gilching, Germany).

MRI and pulse artifact correction were performed based on the average artifact subtraction (AAS) method (Allen et al., 1998) as implemented in Vision Analyzer2 (Brain Products, Germany) followed by objective (CBC parameters, Vision Analyzer) ICA-based rejection of residual artifact-laden components after AAS resulting in EEG with a sampling rate of 250 Hz. Good quality EEG was obtained, which allowed sleep staging by an expert according to the AASM criteria (American Academy of Sleep Medicine, 2007).

### fMRI preprocessing

Using Statistical Parametric Mapping (SPM8) EPI data were realigned, normalized (MNI space) and spatially smoothed (Gaussian kernel, 8 mm^3^ full width at half maximum). The data were band-pass filtered in the range 0.01–0.1 Hz using a sixth order Butterworth filter. The same procedure was applied to the sleep dataset and to the 1000 Functional Connectomes dataset.

### Multivariate classification

We compared the accuracy of a Random Forest classifier with 100 estimators (implemented in scikit-learn, http://scikit-learn.org/stable/) to distinguish younger (< 20 years) and older (>40 years) subjects from the 1000 Functional Connectomes dataset. This was based both on strength and interhemispheric connectivity maps obtained via normalized co-activation matrices (derived from the point-process) and standard linear correlation matrices. We applied a 5-fold cross validation procedure combined with feature selection (*F*-test to retain the top 10, 25, 50, 75% features), as well as with all features. Accuracy was reported as the area under the receiver operator characteristic (ROC) curve (AUC).

## Results

### Correlations between ${\tilde{\text{C}}}_{\text{ij}}$ and R_ij_

**Figure 2:**
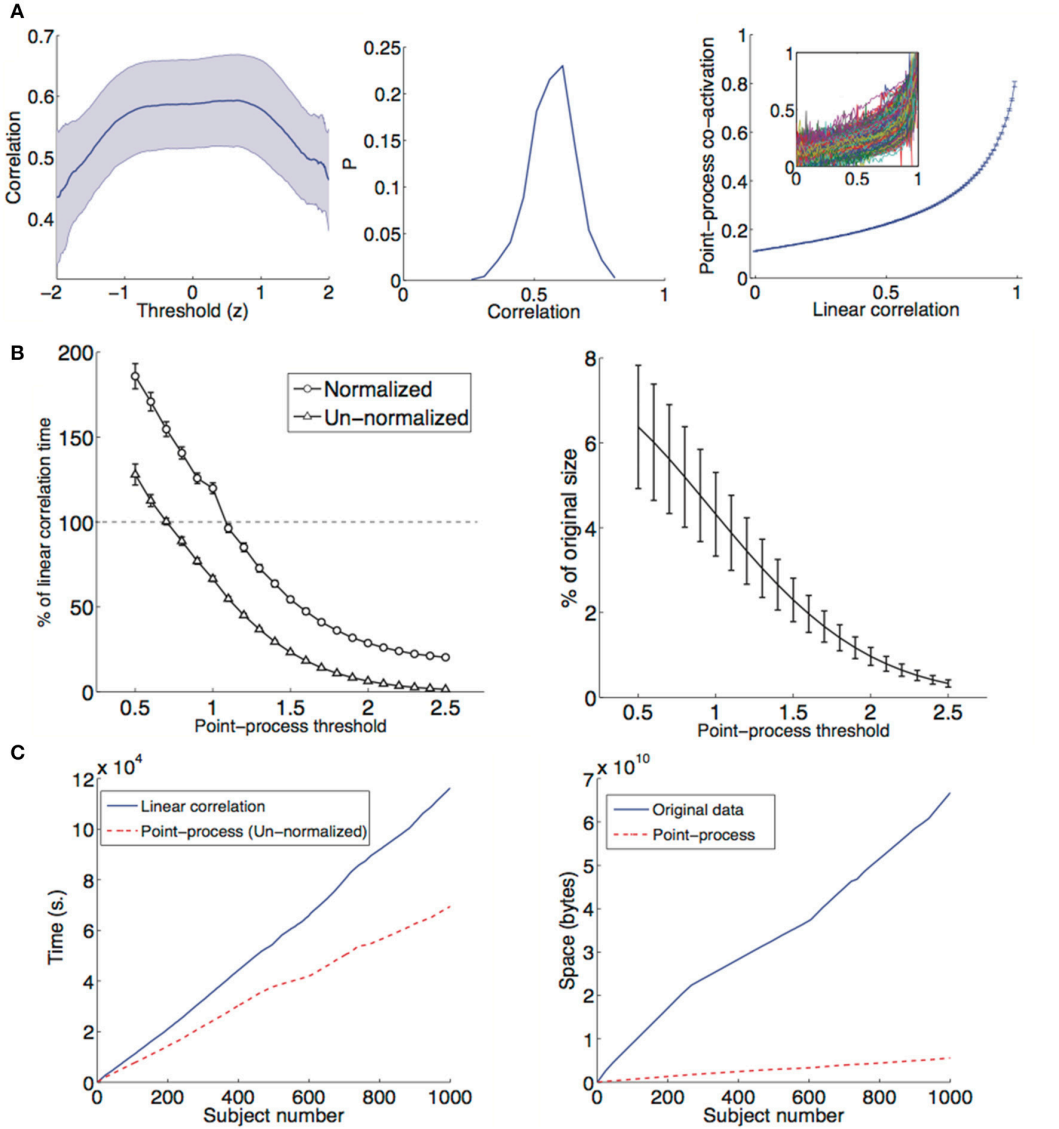
**(A)** Left: Correlation between R_ij_ and ${\tilde{\text{C}}}_{\text{ij}}$ as a function of the threshold [γ in Equation (2); mean ± SEM]. Connectivity networks were derived from 116 time series extracted from the AAL template in all subjects from the 1000 Functional Connectomes dataset (*n* = 1147). Center: Histogram of all correlation values at γ = 1, *P* = probability. Right: Average (mean ± SEM) plot of the linear correlation coefficient between brain regions (entries of R_ij_) and the estimate from the point-process analysis (entries of ${\tilde{\text{C}}}_{\text{ij}}$). The inset shows the plot for each one of the 1147 subjects. **(B)** Left: Performance of the point-process based estimation of functional connectivity as a function of the threshold γ (mean ± SD). Elapsed computation times were obtained for a single subject across 100 repetitions and compared with the performance using linear correlations. Right: Percentage of the original number of data points retained after converting the data to a sparse point-process with γ = 1, plotted as a function of the threshold (for all subjects in the 1000 Functional Connectomes dataset). **(C)** Left: Cumulative computation time required to compute whole-brain voxel-wise connectivity matrices from 1000 subjects extracted from the Functional Connectomes dataset. An un-normalized point-process with γ = 1 was used. Right: Cumulative space required to store 1000 subjects from the Functional Connectomes dataset, both for the full data and for a sparse representation based on a point-process with γ = 1.

We obtained the point-process for both datasets following the procedure illustrated in Figure 1 and in the methods section. In the case of the 1000 Functional Connectomes dataset we repeated calculations both for voxel-wise networks and for networks based on time series extracted from the AAL template. Using this data, we first evaluated the similitude in the estimation of the connectivity matrix by both methods (point-process analysis with normalization and linear correlations) as a function of the threshold γ used to define the point-process (see Equation 2). Results are shown in Figure 2 (left) for the average correlation between connectivity networks estimated by both methods as a function of γ. Correlations peaked at 0.6 and were highest for ≈ 0.7. The histogram of all 1147 correlations obtained using γ = 1 (Figure 2, center) revealed a sharp peak around the mean value. The plot of the entries of the estimated correlation (values of ${\tilde{\text{C}}}_{\text{ij}}$) and the linear correlation (entries of R_ij_) is shown in Figure 2 (right). A monotonously increasing relationship was present between both quantities, even though the functional dependency between them was not linear. For low linear correlation values, the point-process co-activation increased slowly and did so more quickly for larger linear correlation values.

We compared the performance of computing voxel-wise functional connectivity matrices using the proposed point-process based method vs. standard linear correlations. In Figure 2B, left, the percentage of the time required using linear correlations (*corrcoef.m* MATLAB function, average time 131.48 s on a reference system) was plotted as a function of the threshold. At every threshold value a total of 100 iterations were performed for a single subject and results were then averaged. For thresholds larger than approximately 1 standard deviation, the point-process based method slightly outperformed the standard computation, with performance becoming increasingly better as the threshold was increased and less points were included in the analysis. However, more evidence needs to be gathered to confirm that the method outperforms the standard linear correlation approach, considering that the routines have not been properly optimized. In Figure 2B (right) we plot the percentage of data points retained after conversion to the point-process. Even for the smallest threshold values, only about 6% of the data was retained. Thus, this very sparse representation of fMRI data contained sufficient information to capture all the differences during deep sleep and in the 1000 Functional Connectomes dataset (see below), requiring but a small fraction of the original time series. Specifically, the required information consists of the (discrete) timings of the events in the point-process (i.e., at which volumes the “points” appear).

To gauge the usefulness of our approach in a real setting, we computed the cumulative time and space required to process (i.e., obtain whole-brain voxel-wise connectivity matrices) and store 1000 subjects extracted from the Functional Connectomes dataset. Results are shown in Figure 2C. An un-normalized point-process with a threshold of γ = 1 resulted in a reduction of computation time (reference system) from a total of ≈30 h to ≈19 h. However, we note again that more careful experiments need to be performed to compare the time performance of both methods.

**Figure 3:**
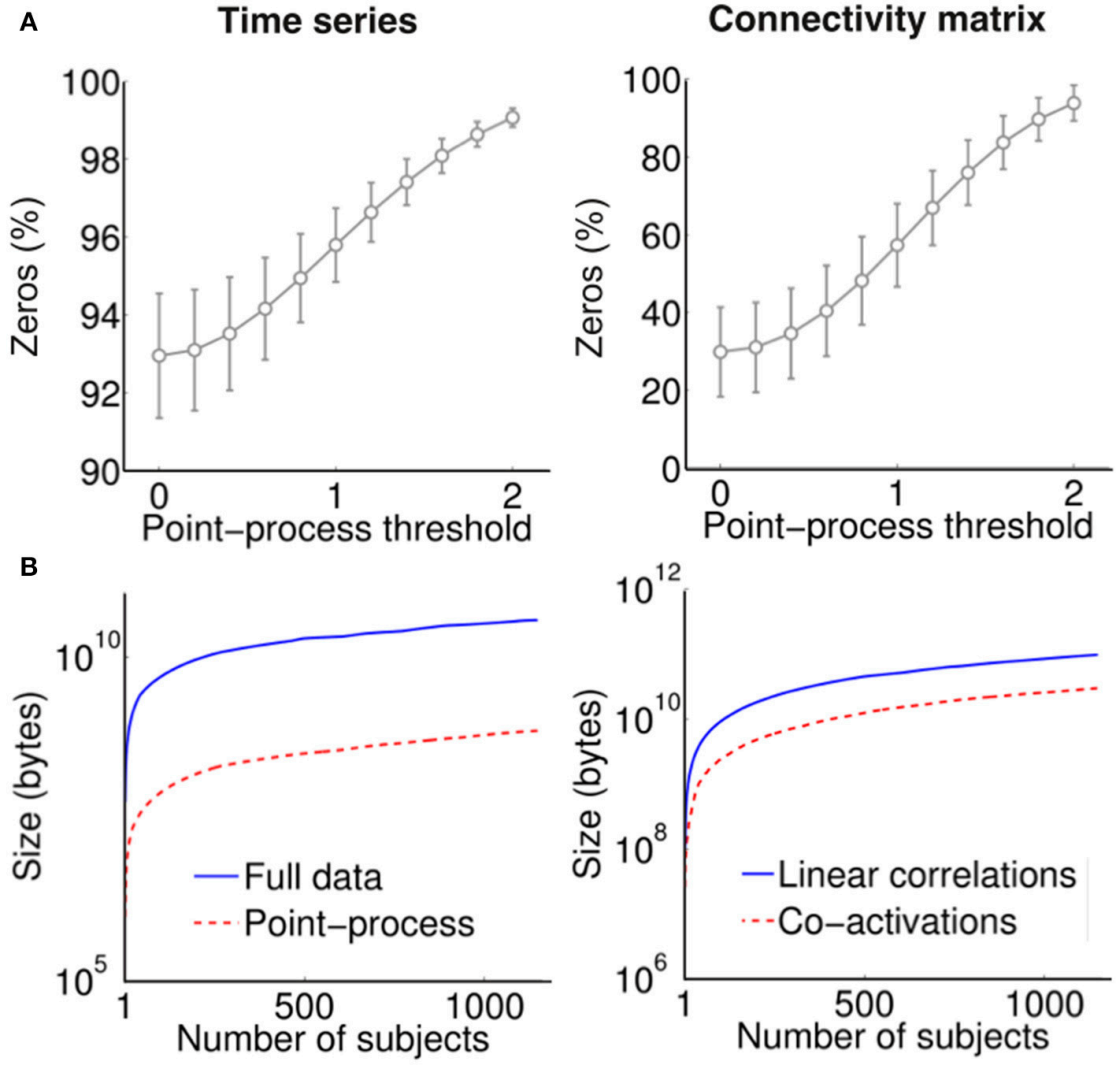
**(A)** Sparseness (% of zero entries) in the time series (left) and connectivity matrices (right) derived using the point-process for a range of thresholds. **(B)** Cumulative file size (in bytes) of fMRI time series (left) and pair-wise connectivity matrices (right) derived using linear correlations (from the full data) and co-activations (from the point-process with threshold equal to 1).

We also investigated the sparseness (defined as the percentage of zeros) in the point-process time series and in the associated normalized connectivity matrices (derived via point-process co-activations). The results are shown in Figure 3A. Not only the time series are very sparse (≈95% zeros for a threshold of 1 S.D.) but also the connectivity matrices (≈50% zeros for the same threshold). This results in dramatically smaller file sizes when both the time series and the connectivity matrices are stored (Figure 3B).

### Strength maps in wakefulness vs. deep sleep

**Figure 4:**
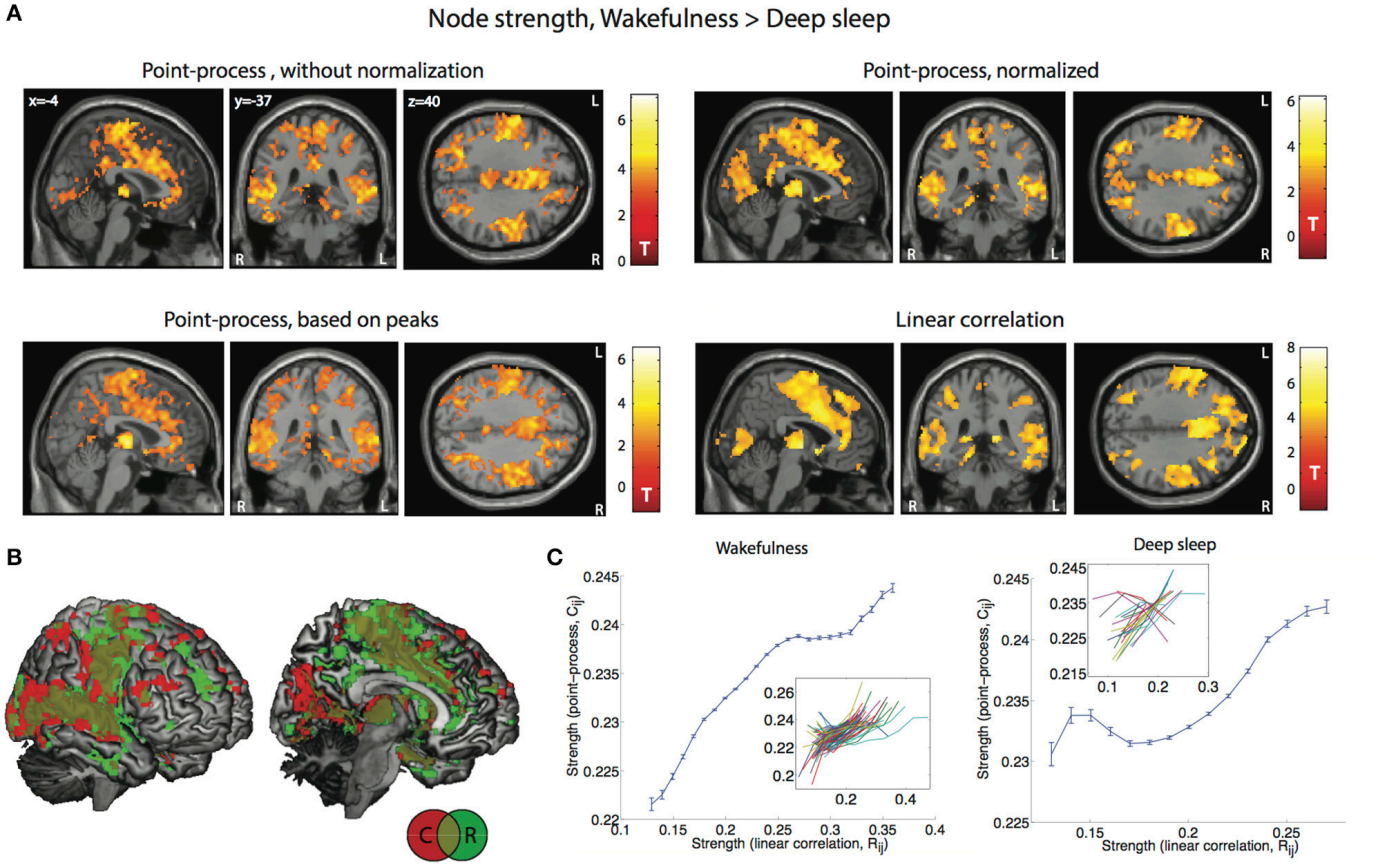
**Voxel-wise changes in node strength can be equally observed from R_**ij**_ and from ${\tilde{C}}_{ij}$**. **(A)** Spatial maps showing voxels with decreased strength in deep sleep (N3 sleep) vs. wakefulness, both for the point-process analysis (un-normalized and normalized), for the peak-based point-process and for linear correlations (display at *p* < 0.05, FWE cluster corrected). **(B)** 3D rendering of the maps in **(A)**: Node strength based on the normalized point-process (red), on linear correlations (green), and their intersection (brown). **(C)** Plot of the node strength values derived from the point-process vs. those derived from the linear correlation (mean ± SEM), for wakefulness (left) and for deep sleep (right). Insets show the results for individual sleep epochs.

To compare results obtained by both methods, we applied them to derive the strength maps (Equation 8) from the estimated whole brain voxel-wise correlations in the sleep dataset and to reveal changes between wakefulness and deep sleep. A total of 20 2-min epochs of deep sleep and 84 epochs of wakefulness could be extracted. After deriving the correlation networks, Equation (8) was applied to obtain the voxel-wise spatial distribution of strengths. Results for the contrast wakefulness > deep sleep are shown in Figure 4A, both for normalized and un-normalized co-activation matrices, as well as for the point-process derived from BOLD signal peaks instead of threshold crossings. Spatial patterns of decreased strength in deep sleep (comprising frontal, cingulate, primary visual, motor, and auditory cortices) were captured equally well by both methods, as well as by the peak-based point-process. In particular, since fixed epoch lengths were used (250 volumes) results were reproduced with and without normalization of connectivity matrices as derived from the point-process. This similitude can also be seen in Figure 4B, in which a joint 3D rendering of both maps shows their spatial agreement. The main plots in Figure 4C show node strength values at all voxels computed using the point-process method (entries of ${\tilde{\text{C}}}_{\text{ij}}$) vs. those computed using linear correlations (entries of R_ij_). The functional dependency was clearly monotonously increasing on average, both for wakefulness and sleep, although two individual epochs of sleep displayed an opposite trend.

### Strength maps in young vs. older subjects

**Figure 5:**
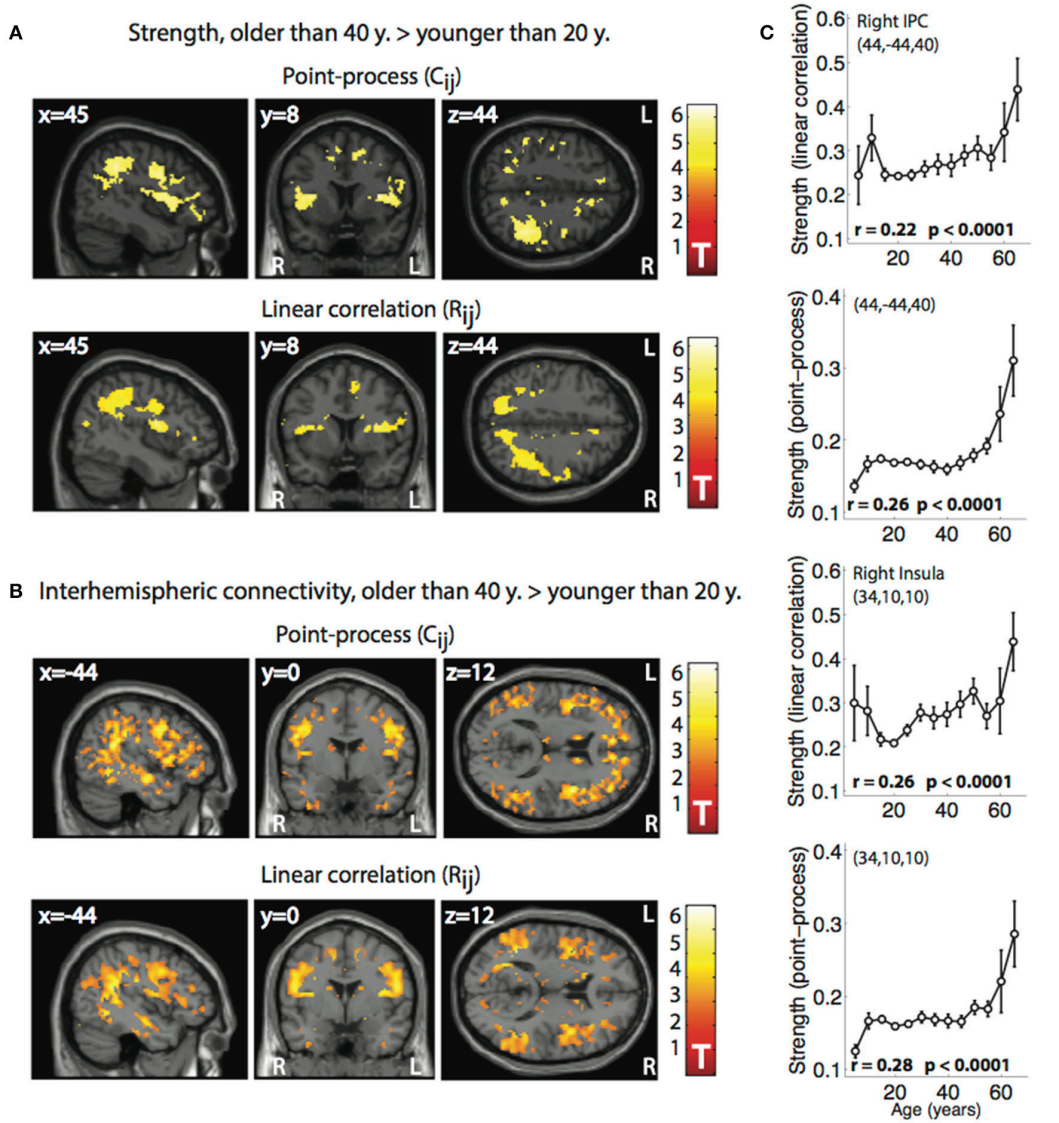
**Voxel-wise changes in node strength and interhemispheric connectivity between two age groups, < 20 years (n = 140) and >40 years (***n*** = 46) observed from R_**ij**_ and from ${\tilde{C}}_{ij}$**. **(A)** Spatial maps showing voxels with increased strength in the older group when compared to the younger group, both for the normalized point-process (${\tilde{C}}_{ij}$, top) and for linear correlation (R_ij_, bottom). Only voxels passing a threshold of *p* < 0.05 (FWE corrected) are shown. **(B)** Spatial maps showing voxels with increased interhemispheric connectivity in the older group when compared with the younger group, both for results obtained from the normalized point-process (${\tilde{C}}_{ij}$, top) and for linear correlation (R_ij_, bottom). Only clusters passing a threshold of *p* < 0.05 (FWE corrected) are shown. **(C)** Plots subject of age (in years) vs. strength values (derived from linear correlations and the normalized point-process) extracted from two regions of interest (right Inferior Parietal Cortex—IPC, and right insular cortex; mean ± SEM). An almost monotonous (but clearly non-linear) relationship between age and network centrality is observed.

We then studied changes in node strength in the 1000 Functional Connectomes dataset, in particular, we compared a group of subjects younger than 20 years with an older group of subjects older than 40 years. Results can be found in Figure 5A. For both methods an increase of (normalized) functional connectivity strength in the older group was observed, comprising a network of regions that included the right parietal cortex, inferior frontal cortex, insula, and the precentral and postcentral gyrus.

Driven by the asymmetry observed in the strength differences between age groups, and by the proposal that the right hemisphere shows accelerated functional decline with aging (Dolcos et al., 2002), we applied linear correlations and the point-process analysis to quantify interhemispheric or homotopic connectivity between groups and compared the respective values. Results are shown in Figure 5B. Increased interhemispheric connectivity was observed for the older group of subjects by both methods, comprising areas in the parietal and temporal cortex, as well as in the precentral gyrus.

Finally, an additional calculation was performed to allow for further evaluation of our method. We regressed subject age vs. strength values in two regions of interest extracted from the analysis of young vs. older subjects (right Inferior Parietal Cortex—IPC, right and insular cortex). Strength values were obtained both from connectivity matrices obtained with linear correlations and with the point-process. Results are shown in Figure 5C. The plots show a moderate increase in strength with age, which suddenly increased for more mature subjects (age > 40 years approximately). Spearman's rank correlation coefficients were higher for the strength values computed using the point-process.

### Classification of young vs. older subjects

**Figure 6:**
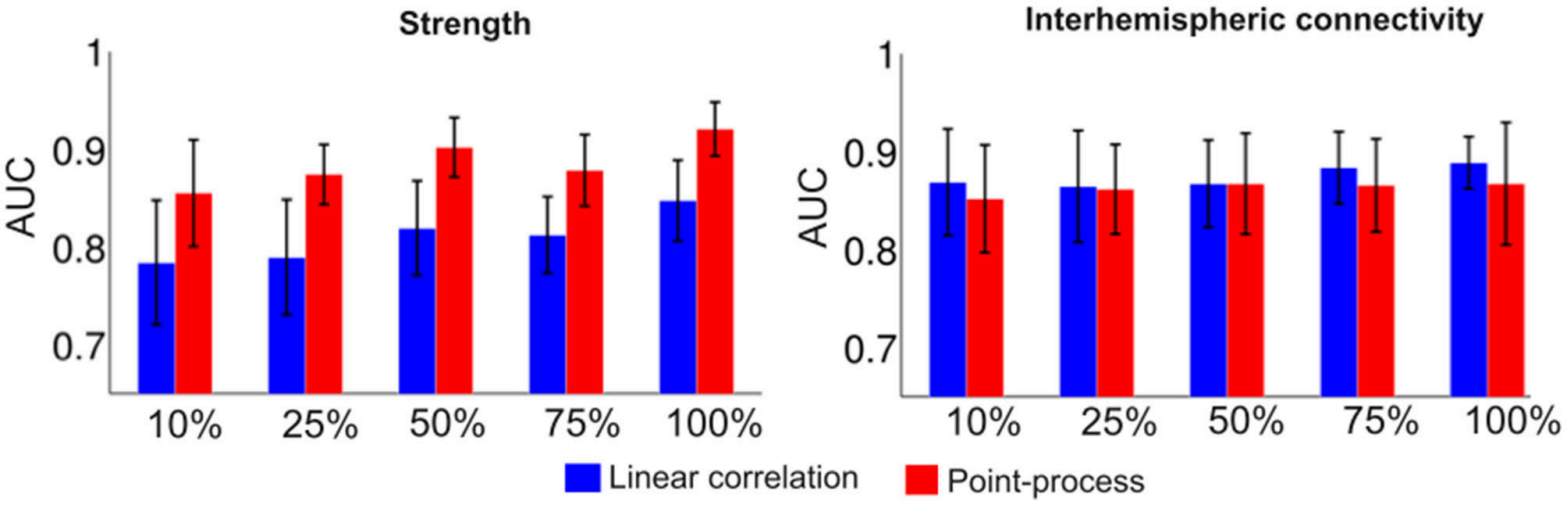
**Classification accuracy of young vs. older subjects based on strength maps and interhemispheric connectivity maps computed using standard linear correlations (blue) and the point-process approach (red)**.

We implemented the classifier described in the methods to investigate how accurately subjects could be classified by age using strength and interhemispheric connectivity maps, computed with both linear correlations and normalized point-process co-activations. Results are presented in Figure 6. We observed similar classification accuracy for the computation based on inter-hemispheric connectivity, and higher classification accuracy for point-process co-activations vs. linear correlations for the computation based on strength maps.

## Discussion

We are witnessing in recent times how neuroscience, and in particular neuroimaging, is moving at a fast pace toward the accumulation and analysis of very large volumes of data. A number of international collaborations is aiming to break new ground in the scale and speed of data collection, including the 1000 Functional Connectomes Project (Biswal et al., 2010), the NIH BRAIN Initiative (Insel et al., 2013), as well as the Human Connectome Project (Van Essen et al., 2013). These studies span hundreds of subjects scanned at high temporal resolution, resulting in very large datasets. Exploratory analyses of this data may thus benefit from biologically principled dimensionality reduction.

While it is obvious that having large volumes of data reduces the negative effect of noise, artifacts and the relative importance of the mathematical models employed to analyze it [a position eloquently defended by Halevy et al. (2009) in their seminal article “The Unreasonable Effectiveness of Data”], it is also true that the handling of redundant data might may be inefficient, both from a computational perspective and in terms of distinguishing the real contributors to the signal from sources of noise. In this line of thought, we have shown that the introduction of a sparse representation of fMRI datasets can reproduce findings obtained from full time series while keeping on the order of 1% of the original data. With respect to vulnerability to noise, sudden head movements can induce spurious points in the process, however, these can be identified from the realignment parameters and erased, following the strategy of scan censoring (Siegel et al., 2014) but eliminating single points (instead of continuous segments of data) from the analyses (see Tagliazucchi et al., 2014 for an application). A consequence of defining the point-process based on high amplitude excursions of the signal is that the impact of physiological noise sources affecting low amplitude fluctuations (Cordes et al., 2002) will be lessened.

### Sleep validation dataset: loss of connectivity in the thalamus, frontal, midline, and auditory cortices

We validated our method by first computing correlation between connectivity matrices as obtained by both methods over > 1000 subjects in the Functional Connectomes dataset, as well as by comparing voxel-wise network strength (a measure of centrality computed from the voxel-wise network of functional connections) between wakefulness and deep sleep and between two age groups extracted from the 1000 Functional Connectomes dataset. In this latter dataset we also obtained the distribution of voxel-wise inter-hemispheric connectivity. The maps of altered network strength in deep sleep and the age-dependent effect observed in the 1000 Functional Connectomes dataset are of biological relevance themselves, as we are not aware of prior reports of these results. Deep sleep resulted in a loss of connectivity across all voxels located in frontal and cingulate cortices, as well as in the primary auditory cortex (Heschl's gyrus) and the thalamus. These are plausible correlates of reduced awareness (frontal and cingulate cortex) and loss of sensory engagement with the environment (primary auditory cortex and thalamus) resulting in increased arousal thresholds (Tagliazucchi et al., 2013).

### Age groups validation dataset: increased connectivity with age in inferior parietal and (pre-)frontal cortices

With respect to the two different age groups extracted from the 1000 Functional Connectomes database, regions central to working memory processes (inferior parietal and frontal cortices, prefrontal cortex) showed “over-connectivity” in the older group of subjects. The meaning of this result is less clear, especially in the light of reports showing an inverse relationship between seed-based functional connectivity and age (Sambataro et al., 2010). However, voxel-based strength maps do not require any *a priori* anatomical hypotheses (i.e., seed selection) and thus might be capable of capturing more global changes in connectivity as opposed to the aforementioned approach. Interestingly, changes in the node strength values were mostly located in the right hemisphere. It has been noted by Dolcos et al. (2002) that the right hemisphere shows a more marked decline with aging, a fact supported so far by evidence from working memory neuroimaging experiments. The changes observed by the authors were hypothesized to be of compensatory origin, which is compatible with the outcome of our analyses (increased overall connectivity in the right hemisphere of older subjects). Prompted by this observation, we also found differences in interhemispheric connectivity located in a set of regions overlapping with those involved with changes in node strength.

### Why few points are sufficient to reproduce functional connectomes

It is worthwhile discussing the reasons underlying the effectiveness of our approach, since it might be surprising that a small fraction of the data suffices to capture all bivariate relationships between BOLD signals (functional connectome) without sacrificing (and even enhancing) classification accuracy.

From a signal processing perspective the answer is relatively straightforward: keeping large amplitude events can increase the signal-to-noise ratio, since it discards low-amplitude activity containing a larger noise component. This non-linear filtering selectively amplifies the importance of those time points at which the signal amplitude becomes relatively large and therefore the signal-to-noise ratio increases. Physiological artifacts have been shown to affect BOLD signals at low frequencies and low amplitudes (Cordes et al., 2002) and signals measured in white matter and cerebrospinal fluid (which do not reflect activity of neural origin and are commonly employed as proxies for physiological confound time series) present smaller amplitude fluctuations compared to those in gray matter (see for instance Tagliazucchi et al., 2013). This situation can result in selective down-weighting of physiological noise when only large-amplitude excursions of the signals are considered.

From a biological point of view, the challenge is to understand why the fMRI time series can be effectively represented as a train of discrete impulses, a view of BOLD time series also supported by studies performing blind de-convolution of spontaneous activity (Petridou et al., 2013). Electrophysiological experiments reveal that Local Field Potentials (LFP) are spatio-temporally distributed as power law avalanches (Beggs and Plenz, 2003): most frequently, spontaneous LFP increases span a limited spatial area, however, at certain (discrete) points in time, LFP might extend up to the size of the tissue under study (an event termed avalanche). If LFP avalanches are, indeed, distributed following a scale-free power law, then macroscopic events (i.e., in the centimeter scale) should be observed, which would be sufficient to elicit a measurable hemodynamic response (considering the correlation observed between LFP and BOLD signals, see Logothetis et al., 2001). Indeed, spatio-temporal avalanches of activity can also be observed with fMRI, following the same statistical laws as the electrophysiological avalanches (Tagliazucchi et al., 2012a). Large amplitude macroscopic LFP increases were reported in the monkey cortex (Thiagarajan et al., 2010) and termed coherence potentials. These large-scale events are also stereotypical (in the words of the authors, much like action potentials at the single-cell level) and thus fulfill all the theoretical requirements for the electrophysiological underpinnings of the events in the spatio-temporal fMRI point-process.

### Contributors to the resting state fMRI signal

One of the main limitations of fMRI compared to other non-invasive neuroimaging techniques (EEG, MEG) is its limited temporal resolution. This limitation not only stems from the relatively slow acquisition of whole-brain volumes (i.e., long TRs, in the order of seconds) but also from the coupling between neural activity and the signal measured by fMRI. This coupling blurs temporally localized activity into a temporally extended response (given by the HRF). Therefore, improvement in fMRI sampling rates will only result in a better-sampled HRF, with no gain in the measurement of underlying neural activity, unless the distortion caused by the HRF can be inverted.

Our results suggest that the fMRI resting state signal comprises a temporal succession of well-localized events. The identification of these events has been shown to match a formal de-convolution of fMRI time series (Tagliazucchi et al., 2012a; Petridou et al., 2013). This inversion of the HRF blurring can allow to capitalize on improvements in fMRI acquisition rates. While the contributors to the task-evoked fMRI signal have been thoroughly investigated, this remains to be done in the context of spontaneous brain activity; the possibility of reducing resting state fMRI signals to a few high-amplitude events and still estimate all pair-wise interactions represents an important first step in this direction, and suggests a focus for future studies on the electrophysiological basis of spontaneous fMRI fluctuations.

### Caveats and limitations

Generally, this procedure should yield equivalent results for any dataset in which high amplitude events do not arise spuriously as artifacts and represent important information in the data. From a neurophysiological perspective, the fulfillment of these conditions has been already demonstrated for BOLD time series by means of inverting the Hemodynamic Response Function (HRF) convolution of neuronal sources (de-convolution). As discussed in the previous sections, LFP giving rise to metabolic changes reflected in the BOLD signal are temporally cluttered into avalanches of activity (Beggs and Plenz, 2003; Tagliazucchi et al., 2012a; Shriki et al., 2013), presumably underlying the high information content of BOLD signal high amplitude events.

The main drawbacks of the proposed method are: (1) the non-linear relationship between linear correlation and its estimated value using the point-process (i.e., point-process co-activation, Figure 2C) and (2) the slowing down of the computation time when following the normalization given by Equation (6), unless properly optimized. With respect to the first concern, while not linear, the relationship is clearly monotonic and by extracting its functional form, connectivity estimated using the point-process can be properly normalized to have a linear co-variation with standard functional connectivity. This non-linear shape can be explained by the dismissal of low amplitude events in the point-process and their associated contributions to linear correlations. Therefore, correlations can increase faster than point-process co-activations, giving rise to the convex shape seen in Figure 2A, right panel. The second concern (normalization) does not affect the results unless performing comparisons between time series of different length, thus having a different number of points. Normalizing by the length of the time series offers a solution to this issue.

### Related findings

Given the relative novelty of the present approach, caution should be exercised concerning the interpretation of the results to avoid making exaggerated claims. Nevertheless, it is encouraging and reassuring to see a body of publications consistent with the main idea of the present paper. Indeed, since the first observation (Tagliazucchi et al., 2012a) that the timing of high-activity events in BOLD signals allows the reconstruction of major RSN, different research groups have reproduced and built on this result (Davis et al., 2013; Liu and Duyn, 2013; Liu et al., 2013; Amico et al., 2014; Jiang et al., 2014; Li et al., 2014). The analysis of spontaneous voxel co-activation is a natural continuation of functional connectivity studies: instead of asking whether two voxels are engaged in synchronized fluctuations over a relatively long period of time, the question is shifted to whether two voxels become jointly activated (i.e., present high activity above their baseline levels) and what are the timings and properties of these co-activations. Interestingly, it has been shown that co-activation patterns contain additional information not available to standard functional connectivity analyses (Liu et al., 2013) and has also been used to characterize the dynamics of different brain states (Amico et al., 2014; Chen et al., 2015). In the present report we show that the spatio-temporal point-process extracted from whole-brain BOLD signals suffices to estimate all pairs of functional connections (i.e., the functional connectomes) with reasonable accuracy (as demonstrated by its usefulness to capture differences in connectivity between brain states/groups of subjects) with a very small fraction of the data (on the order of 1%), and thus can be taken as an equivalent (but sparser) representation of the data. We believe these results should prompt an in-depth exploration of high amplitude events in BOLD time series, in particular, their neural correlates and potential relationship to LFP neural avalanches, a signature of self-organized criticality in the human brain (Chialvo, 2010).

In conclusion, as fMRI datasets grow larger, tools to rapidly store, process, and explore them become increasingly valuable. The present report validates a strategy defining a sparse representation of these complex four-dimensional datasets, which keeps only the timing of large BOLD events and thus allows for reasonable fMRI compression. This technique both empowers neuroimaging collaborative projects aimed at gathering and understanding vast amounts of data, and suggests a temporally intermittent organization for brain hemodynamic activity, likely reflecting discrete electrophysiological events spreading throughout the cerebral cortex. Vice versa, if we assume that the sub-threshold BOLD activity is not mere noise nor redundant, this reminds us that with functional connectivity analyses we take but a peek through a keyhole onto the wealth of brain function.

## Author contributions

All authors listed, have made substantial, direct, and intellectual contribution to the work, and approved it for publication.

### Conflict of interest statement

The authors declare that the research was conducted in the absence of any commercial or financial relationships that could be construed as a potential conflict of interest. The reviewer JV and handling Editor declared their shared affiliation, and the handling Editor states that the process nevertheless met the standards of a fair and objective review.
